# Impact of the SCF signaling pathway on leukemia stem cell-mediated ATL initiation and progression in an HBZ transgenic mouse model

**DOI:** 10.18632/oncotarget.10210

**Published:** 2016-06-21

**Authors:** Wakako Kuribayashi, Kazuya Takizawa, Kenji Sugata, Madoka Kuramitsu, Haruka Momose, Eita Sasaki, Yuki Hiradate, Keiko Furuhata, Yoshihisa Asada, Atsushi Iwama, Masao Matsuoka, Takuo Mizukami, Isao Hamaguchi

**Affiliations:** ^1^ Department of Safety Research on Blood and Biological Products, National Institute of Infectious Disease, Tokyo, Japan; ^2^ Department of Cellular and Molecular Medicine, Graduate School of Medicine, Chiba University, Chiba, Japan; ^3^ Faculty of Pharmaceutical Sciences, Tokyo University of Science, Chiba, Japan; ^4^ Laboratory of Virus Control, Institute for Virus Research, Kyoto University, Kyoto, Japan

**Keywords:** ATL, HBZ, leukemia stem cells, cancer stem cell, SCF

## Abstract

Adult T-cell leukemia (ATL) is a malignant disease caused by human T-lymphotropic virus type 1. In aggressive ATL, the response to chemotherapy is extremely poor. We hypothesized that this poor response is due to the existence of chemotherapy-resistant cells, such as leukemic stem cells. Previously, we successfully identified an ATL stem cell (ATLSC) candidate as the c-kit^+^/CD38^−^/CD71^−^ cells in an ATL mouse model using Tax transgenic mice. Here, with a new ATL mouse model using HBZ-transgenic mice, we further discovered that the functional ATLSC candidate, which commonly expresses c-kit, is drug-resistant and has the ability to initiate tumors and reconstitute lymphomatous cells. We characterized the ATLSCs as c-kit^+^/CD4^−^/CD8^−^ cells and found that they have a similar gene expression profile as T cell progenitors. Additionally, we found that AP-1 gene family members, including *Junb*, *Jund,* and *Fosb,* were up-regulated in the ATLSC fraction. The results of an *in vitro* assay showed that ATLSCs cultured with cytokines known to promote stem cell expansion, such as stem cell factor (SCF), showed highly proliferative activity and maintained their stem cell fraction. Inhibition of c-kit–SCF signaling with the neutralizing antibody ACK2 affected ATLSC self-renewal and proliferation. Experiments in Sl/Sld mice, which have a mutation in the membrane-bound c-kit ligand, found that ATL development was completely blocked in these mice. These results clearly suggest that the c-kit–SCF signal plays a key role in ATLSC self-renewal and in ATL initiation and disease progression.

## INTRODUCTION

Adult T-cell leukemia (ATL) is a peripheral T cell leukemia caused by infection with human T-lymphotropic virus type 1 (HTLV-1) [1, 2]. HTLV-1 infection mainly occurs through breast-feeding, and after a long latency period, HTLV-1 infected cells finally develop into ATL cells [3]. Most infectious carriers never show any clinical signs, but approximately 3-5% of HTLV-1 carriers develop ATL [4]. Although combination chemotherapy has been promising in patients with indolent type ATL, the prognosis is much worse for patients with the most aggressive ATL type, with frequent resistance to or early relapse after intensive polychemotherapy [5, 6].

The existence of chemotherapy- and radiation therapy-resistant cancer stem cells (CSCs) [7], which are characterized by tumor initiating ability, self-renewal, and drug resistance and are a phenotypically and molecularly distinct rare population that can reproduce original tumor cell types in *in vivo* transplantation assays, has been hypthesized [8]. The CSC hypothesis is supported experimentally by findings from some hematological malignancies [9–13] and solid tumors [14, 15]. These findings provide strong evidence that CSCs might have a key role in cancer development and chemotherapy resistance.

Recent studies suggest that ATL cells are phenotypically [16, 17], functionally, and molecularly heterogeneous [18]. Indeed, using criteria that CSCs harbor a high dye efflux function associated with drug resistance [19, 20], we found a functional ATL stem cell (ATLSC) candidate in an ATL mouse model using Tax-transgenic (Tax-Tg) mice [21, 22]. El Haji *et al*. also reported a tumor initiating ability in ATL cells from a Tax-Tg ATL mouse model, suggesting that these cells could be a therapeutic target for ATL [23]. More recently, Nagai *et al*. reported that T memory stem cells are responsible for the hierarchical apex of ATL that can reproduce patient ATL types in recipient immunodeficient mice [24]. These data strengthen the idea that rare ATLSCs might play a key role in ATL development and progression. However, it is still unclear what common feature of ATLSCs could be used as a molecular therapy target and which HTLV-1 factor and genetic pathway cause ATLSC development and regulation.

Here, we show strong evidence that ATLSCs exist in another mouse model of ATL using HBZ-transgenic mice (HBZ-Tg). Although HTLV-1 Tax transcripts are only detected in about 60% of ATL cases [25, 26], the HTLV-1 b-zip factor (HBZ) gene is expressed in all ATL cases, and HBZ induced ATL/lymphoma development *in vivo* [27]. We also report that a common surface marker of ATLSCs, c-kit, is a key regulator of ATL disease initiation and progression. Thus, our findings support the ATLSC hypothesis and indicate that c-kit-SCF (stem cell factor) signaling could be a therapeutic target for ATL.

## RESULTS

### HBZ-expressing mouse ATL cells possess tumor initiating ability

**Figure 1:**
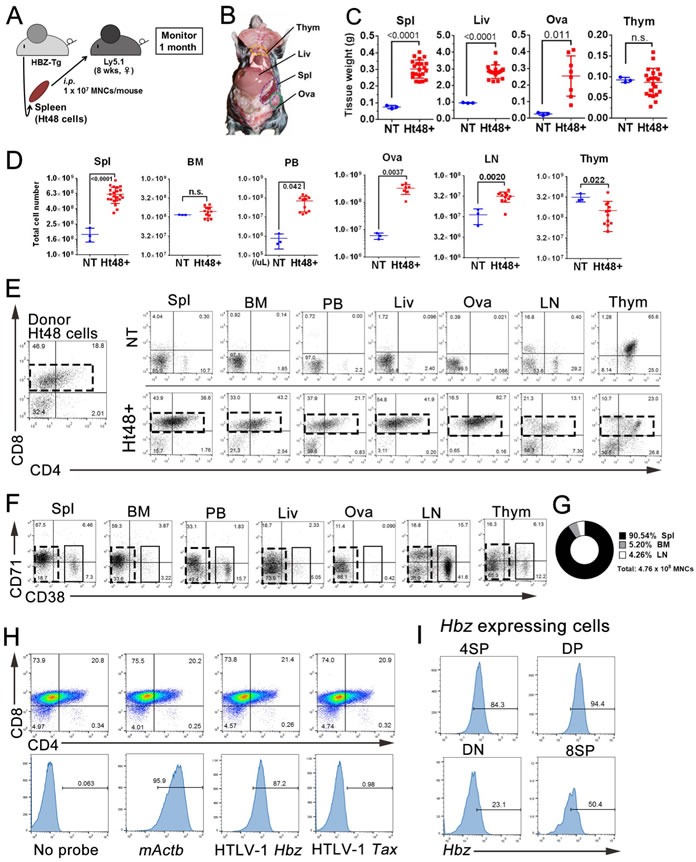
Assessment of the *in vivo* tumor initiating ability of HBZ-expressing mouse ATL cells (Ht48) **A.** Schematic representation of this experiment. We transplanted 1×10^7^ Ht48 cells derived from HBZ-Tg mouse splenic lymphomatous cells intraperitoneally (*i.p.*) into C57BL/6 (Ly5.1) mice. **B.** The gross anatomy of the recipient mouse 20 days after Ht48 cell transplantation. **C.**-**D.** Graphs depicting the weight **C.** and the cell number **D.** of each tissue after Ht48 cell transplantation. Each mouse is represented by a dot. Horizontal lines indicate the median. NT, no transplant; Ht48+, Ht48 cell transplantation; n.s., not significant. **E.**-**F.** Flow cytometric analyses to detect CD4 and CD8 expression **E.** or CD71 and CD38 expression **F.** in donor Ht48 cells from a recipient mouse. Representative flow cytometry plots of Ht48 cells before (left) and after transplantation (right) are shown. Boxes composed of red **E.** and **F.** or black **F.** dashed lines indicate the major Ht48 population. **G.** Graph depicting the percentage of the Ht48 cell distribution in each lympho-hematopoietic tissue. **H.** A flow cytometric analysis was performed using a Primeflow™ assay to detect HBZ transcript in Ht48 cells. The Ht48 cells were separated by forward and side scatter (FSC and SSC, respectively) and by CD4^+^ and CD8^+^ for each gene transcript. Representative flow cytometry plots are shown, and the histograms depict the expression level of each gene in Ht48 cells. No Probe, no probe hybridization; ACTB, mouse actin beta. **I.** Representative graphs of the HTLV-1 HBZ expression level in 8SP (CD4^−^CD8^+^), DP (CD4^+^CD8^+^), DN (CD4^−^CD8^−^), or 4SP (CD4^+^CD8^−^) Ht48 cells. Spl, spleen; BM, bone marrow (femur); PB, peripheral blood; Liv, liver; Ova, ovary; LN, lymph node (inguinal and axillary); Thym, thymus; NT, no transplantation.

In this study, we used ATL cells (named Ht48) isolated from an HBZ-Tg mouse [27, 28]. To assess the tumor initiating and regeneration abilities of Ht48 cells *in vivo*, we transplanted 1×10^7^ Ht48 cells into the C57BL/6-Ly5.1 mouse (Ly5.1) strain whose cells can be distinguished from the donor cells by FACS (Figure 1A). As in the donor HBZ-Tg mouse, ATL-like lymphoma in the recipient mouse can be recognized as spleen, liver, and ovary hypertrophy (Figure 1B). All recipient Ly5.1 mice showed body weight loss (data not shown), hepatosplenomegaly, and died within 30 days post-transplantation. The spleen, liver, and ovary weights were significantly increased in the recipient mice compared with those in control mice (no transplant, NT) (Figure 1C). Massive tumor cell infiltration can be seen in the recipient mouse spleen and ovaries. The numbers of MNCs, including lymphoma cells in the peripheral blood (PB), spleen, ovaries, LNs, and thymus, were also significantly higher in recipient mice than in NT mice (Figure 1D).

To characterize the lymphoma cell phenotype in each of the infiltrated tissues, we performed FACS analyses of previously identified surface antigens observed in the HBZ-Tg mouse model. Ht48 cells were classified into CD4 single-positive (4SP), CD8 single-positive (8SP), and CD4/CD8 double-positive or -negative (DP and DN cells, respectively) cells. The majority of the donor Ht48 cells were 8SP, DP, or DN. A similar phenotype to that observed in donor Ht48 cells was also detected in the recipient spleen, BM, PB, ovaries, and liver but not in the recipient LNs or thymus (Figure 1E).

Another ATL cell phenotypes previously reported in the Tax-Tg mouse model of ATL [22], CD38^+^ and CD71^+^, was also evaluated in this HBZ-Tg model. In the major ATL cell population, both CD71^+^ and CD38^+^ cells were detected in the recipient spleen, PB, and BM but were not observed in the recipient ovaries, liver, LN, or thymus (Figure 1F). By using two different phenotypic analyses of mouse ATL cells, both Ht48 cell phenotypes (CD38^+^ and CD71^+^) can be commonly detected in the recipient PB, BM, and spleen. We calculated the average number of Ht48 cells in each of the hematopoietic and lymphoid tissues (Figure 1G) and found that the spleen is the most efficient tissue for Ht48 proliferation.

**Figure 2:**
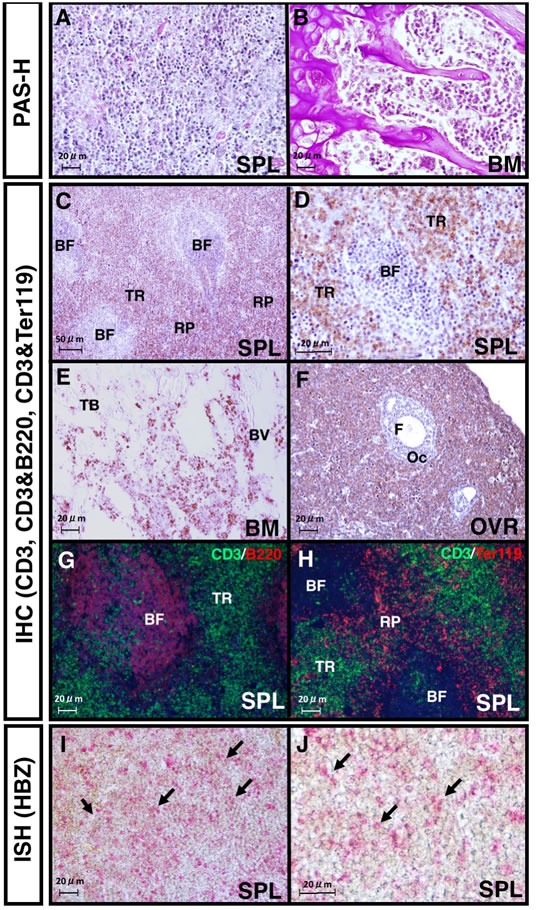
Histological analysis of lymphomas formed in the recipient spleens **A.**, **B.** Images of PAS- and hematoxylin- stained spleen **A.** or bone marrow (BM) sections **B.** 20 days after Ht48 cell transplantation. **C.** CD3-staining image of a section of a lymphoma that formed in a recipient spleen. BF: B follicle zone; TR: T cell-rich zone; RP: red pulp. **D.** High magnification image of the CD3 immunostaining of the spleen. **E.** Image of CD3 staining of a lymphoma-infiltrated recipient BM section. TB: trabecular bone zone; BV: blood vessel. **F.** Image of CD3 staining of a section of lymphoma-infiltrated recipient ovary. OC: oocyte; F: follicle **G.**-**H.** Images from immunofluorescence detections (IHC) of CD3 and B220 or CD3 and Ter119 in a section of a lymphoma that formed in a recipient spleen. **I.**-**J.** Images from an *in situ* hybridization (ISH) analysis of the HBZ transcript levels in a lymphoma that was formed in a recipient spleen. Red dots show HBZ transcript. Arrows indicate HBZ expression in the lymphoma-formed spleen. All images shown are representative of repeated observations. Scale bar: 100 μm.

HBZ expression in recipient mice was also detected by FACS analysis in splenic Ht48 cells (Figure 1H), and it was high in the DP, 8SP, and 4SP subsets (Figure 1I). A similar observation was made in the histological spleen sections. The ATL cells can be seen in the spleen, BM (Figure 2A and 2B), ovaries, and liver (data not shown). CD3^+^ T cells can be observed in the spleen, BM, and ovaries (Figure 2C-2F). In the spleen, CD3^+^ cells were found in the T cell-rich region but not in the B cell-rich follicle (Figure 2G) and near the red pulp (Figure 2H). To identify the splenic ATL cells, we investigated the HBZ expression in spleen sections using *in situ* hybridization. We found that some HBZ-expressing Ht48 cells can be seen in the splenic CD3^+^ cell-rich region (Figure 2I-2J). Together, these findings suggest that the spleen is the major site of ATL cell proliferation *in vivo* and that splenic ATL cells possess tumor initiating capacity, both phenotypically and functionally.

### Ht48 cells with tumor initiating ability act as stem cells *in vivo*

**Figure 3:**
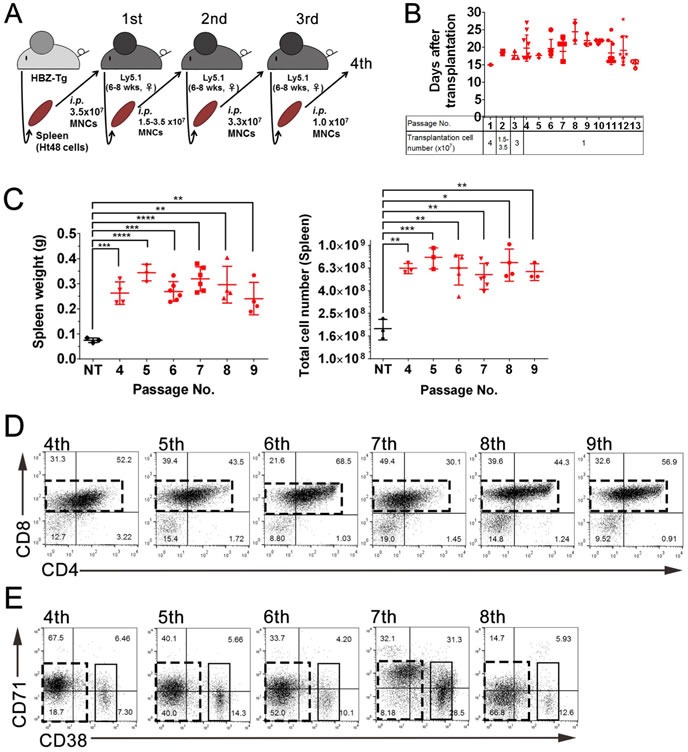
Evaluation of the *in vivo* ATLSC ability of Ht48 cells by a serial transplantation assay **A.** Schematic representation of the consecutive serial transplantation experiment. A total 1-4 ×10^7^ Ht48 cells were transplanted into C57BL/6 (Ly5.1) mice intraperitoneally (*i.p*) for the initial three consecutive serial transplantations, after which we transplanted 1×10^7^ Ht48 cells. wks, weeks **B.** Graph depicting the survival time after each of the 13 consecutive serial transplantations. Each mouse is represented by a dot. Horizontal lines indicate the median survival time. **C.** Graph depicting the spleen weight (left panel) and the number of MNCs in the spleen (right panel) at the sacrifice time points of passages 4-9 in the 13 consecutive serial transplantations experiment. Each mouse is represented by a dot. Horizontal lines indicate the median. NT: no transplantation control. **p < 0.05; ***p < 0.005; ****p < 0.0005. **D.**-**E.** Representative graphs from flow cytometric analyses to detect CD4 and CD8 expression **D.** or CD71 and CD38 expression **E.** in donor Ht48 cells from recipient mice after passages 4-9 **D.** or passages 4-8 **E.** in the 13 consecutive serial transplantations experiment. Boxes composed of red **D.** and **E.** or black **E.** dashed lines indicate the major Ht48 cell population.

To assess the capacity of Ht48 cells to act as stem cells and evaluate their tumor initiating ability, we designed a serial transplantation experiment. For each serial transplantation experiment, we transplanted 1-4×10^7^ Ht48 cells isolated from former recipient lymphomas that developed in Ly5.1 mouse spleens into new recipient mice. We performed thirteen consecutive serial transplantation experiments (Figure 3A). The recipient mice died within 15-25 days in each of the transplantation experiments (Figure 3B). Consistent with the results described above, both the spleen tissue weight and total MNC number were significantly increased in recipient mice compared with those in NT mice (Figure 3C). We also performed FACS analyses to identify if Ht48 cells were capable of regenerating the original tumor phenotype in recipient mice. Similar Ht48 cell phenotypes (4SP, 8SP, DP, DN and CD38^+^/CD71^+^) were observed in the recipient mice following each serial transplantation experiment (Figure 3D and 3E). These data suggest that functional ATLSCs with a self-renewal activity may exist among Ht48 cells.

### A subpopulation of high drug efflux capacity and c-kit expression cells exist in the Ht48 cell population

**Figure 4:**
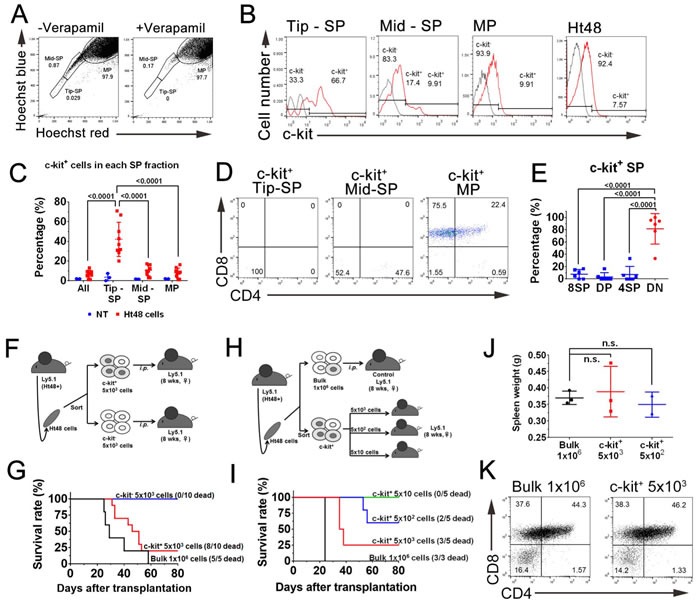
Evaluation of the dye efflux function in ATLSCs by a subpopulation analysis of Ht48 cells **A.** Representative flow cytometric dot plot of the SP analysis of Ht48 cells. The dot plots show control Ht48 cells incubated in Hoechst33342 alone (left) or in the presence of 100 μM verapamil (right). **B.** Representative histograms showing the proportion of c-kit^+^ cells in the Tip-SP, Mid-Sp, and MP fractions and in the unseparated Ht48 cells. **C.** Graph depicting the percentage of c-kit^+^ cells in the SP and MP fractions. **D.** Representative dot plots from a flow cytometric analysis to detect CD4 and CD8 expression in the c-kit^+^ SP donor Ht48 cells. **E.** Graph depicting the percentages of c-kit^+^ SP cells that were 8SP, DP, 4SP, or DN. Each mouse is represented by a dot. **F.** Schematic representation of the c-kit^+^ Ht48 cell transplantation experiment. A total of 5×10^3^ c-kit^+^ or c-kit^−^ Ht48 cells or 1×10^6^ bulk Ht48 cells were transplanted intraperitoneally into C57BL/6(Ly5.1) mice. Ht48+, Ht48 cell transplantation **G.** The overall survival of recipient mice transplanted with 1×10^6^ Ht48 cells, 5×10^3^ c-kit^+^ cells, or c-kit^−^ Ht48 cells. The overall difference in survival between mice transplanted with c-kit^+^ cells or c-kit^−^ cells was statistically significant (*p* < 0.0001). **H.** Schematic representation of the follow-up c-kit^+^ Ht48 cell transplantation experiment. We intraperitoneally transplanted 5×10^1^ to 5×10^3^ c-kit^+^ Ht48 cells or 1×10^6^ bulk Ht48 cells into C57BL/6 (Ly5.1) mice. **I.** The overall survival of recipient mice transplanted with 1×10^6^ bulk Ht48 cells or with 5×10 to 5×10^3^ c-kit^+^ Ht48 cells. The overall difference in the survival between each of the groups was statistically significant (*p* < 0.0001). **J.** Graph depicting the weight of the spleen at the sacrifice time point after the transplantation experiment described above. Each mouse is represented by a dot. Horizontal lines indicate the median value. **K.** Representative dot plots from a flow cytometric analysis to detect CD8 and CD4 expression in unfractionated Ht48 cells and c-kit^+^ Ht48 cells after they were transplanted into recipient mice.

To identify Ht48 cell ATLSC candidates, we performed a SP analysis that has been used previously to identify drug-resistant CSCs in various tumors4. Ht48 cells were divided into three SPs based on their Hoechst33342-low-fluorescence profile: the tip-SP fraction (Tip-SP; 0.029%), the mid-SP population (Mid-SP; 0.87%), and the major population (MP; 97.9%) (Figure 4A). The ABCG transporter inhibitor, verapamil, effectively blocked the emergence of nearly all Tip-SP and most Mid-SP Ht48 cells. We found that > 65% of the Tip-SP cells were ATLSC surface marker, c-kit^+^ while < 10% of the non-SP fraction cells (MPs) were c-kit^+^ (Figure 4B-4C). Approximately 5% of the total Ht48 cell population were c-kit^+^, and of the c-kit^+^ cells, most were 8SP, followed by DP, DN, and 4SP cells (Figure 4D). The c-kit^+^ cells in the Tip-SP and Mid-SP fractions were mainly composed of DN cells (> 70%), whereas the c-kit^+^ MP cells were predominantly composed of 8SP and DP (Figure 4E). These results suggest that the c-kit^+^ ATLSC candidate with a high drug efflux capacity is enriched in the SP fractions and has a DN phenotype.

To assess the tumor repopulation ability of the c-kit^+^ Ht48 cells, we transplanted 5×10^3^ c-kit^+^ or c-kit^−^ cells into Ly5.1 mice (Figure 4F). Whereas the mice that received a 5×10^3^ c-kit^−^ Ht48 cell transplantation lived for the entire observation period (80 days), the mice that received a 1×10^6^ unfractionated Ht48 cell or 5×10^3^ c-kit^+^ Ht48 cell transplantation died at similar rates during this period (Figure 4G). Because approximately 5% of Ht48 cells are c-kit^+^ (Figure 4B), we speculated that 1×10^6^ unfractionated Ht48 cells likely contain 5×10^4^ c-kit^+^ cells. Thus, we next intraperitoneally injected 5×10^1-3^ c-kit^+^ Ht48 cells or 1×10^6^ Ht48 cells as a control into the Ly5.1 mice to assess the c-kit^+^ cell tumor initiating ability (Figure 4H). No mice died after receiving 5×10^1^ c-kit^+^ cells, but over half of the mice (3/5) died after receiving 5×10^3^ c-kit^+^ cells. The survival rate of the recipient mice gradually decreased in a dose dependent manner (Figure 4I). A phenotypic analysis of post-transplantation Ht48 cells revealed that different numbers of transplanted c-kit^+^ cells all caused ATL development like transplanted bulk Ht48 cells (Figure 4J and 4K). Together, these data clearly suggest that the population of c-kit^+^ Ht48 cells may contain functional ATLSCs.

### c-kit^+^ DN cells have *ex vivo* self-renewal capacity *in vivo*

**Figure 5:**
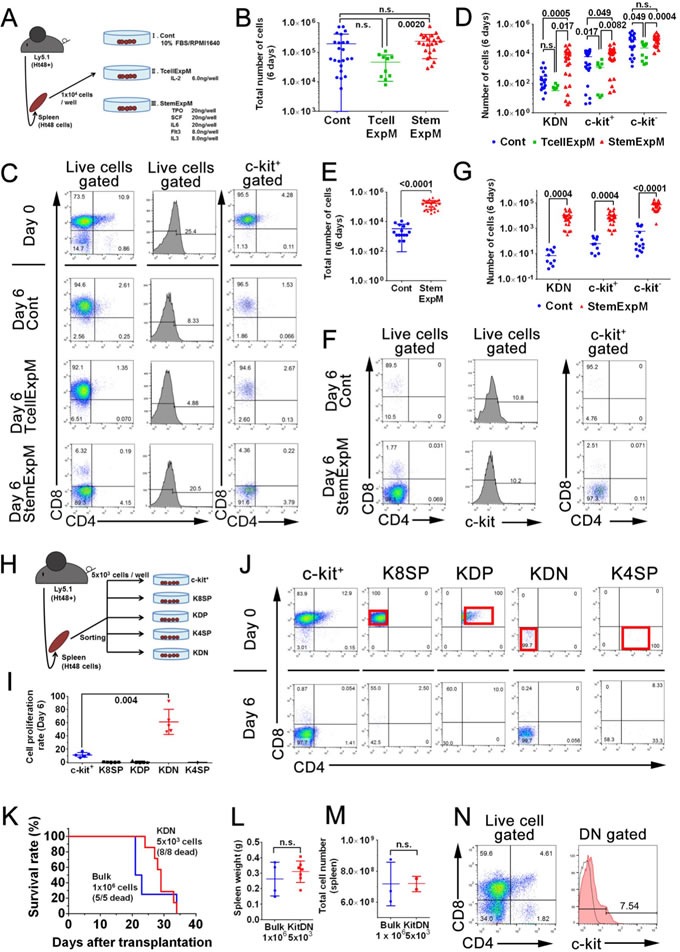
Effect of cytokine presence on ATLSC proliferation and differentiation **A.** Schematic representation of the *in vitro* Ht48 cell proliferation assay. A total of 1×10^4^ Ht48 cells were cultured under multiple culture conditions for 6 days. Control media (Cont) consisted of base media (RPMI1640 with 10% FBS) without cytokines. T cell expansion-inducing medium (TcellExpM) contained 6 ng/well IL-2 in base media. The stem cell expansion-inducing medium (StemExpM) contained five cytokines (20 ng/well TPO, 20 ng/well SCF, 20 ng/well IL-3, 8 ng/well IL-6, and 8 ng/well Flt3) in base media. **B.** Graph depicting the number of MNCs after a 6-day culture under the conditions described in (A). Each culture well is represented by a dot. Horizontal lines indicate the median number of MNCs. **C.** Representative dot plots from a flow cytometric analysis to detect CD4, CD8, and c-kit expression in donor Ht48 cells after a 6-day culture under the culture conditions described in (A). **D.** Graph depicting the number of c-kit^+^CD4/8DN (KDN), c-kit^+^, and c-kit^−^ Ht48 cells after a 6-day culture under the conditions described in A. Each culture well is represented by a dot. Horizontal lines indicate the median number of cells. **E.** Graph depicting the number of MNCs after a 6-day culture of c-kit+ Ht48 cells under the conditions described in (A). Each culture well is represented by a dot. Horizontal lines indicate the median number of MNCs. **F.** Representative dot plots from a flow cytometric analysis to detect CD4, CD8, and c-kit expression in Ht48 cells after a 6-day culture of c-kit^+^ Ht48 cells under the conditions described in (A). **G.** Graph depicting the number of KDN, c-kit^+^, and c-kit^−^ Ht48 cells after a 6-day culture under the conditions described in (A). Each culture well is represented by a dot. Horizontal lines indicate the median number of cells. **H.** Schematic representation of the *in vitro* Ht48 cell proliferation assay. Ht48 cells were subdivided and sorted as a c-kit^+^CD4^−^CD8^+^ (K8SP), c-kit^+^CD4^+^CD8^+^ (KDP), c-kit^+^CD4^+^CD8^−^ (K4SP), and c-kit^+^CD4^−^CD8^−^ (KDN) cells. A total of 1×10^4^ of each type of cells was cultured under StemExpM for 6 days. **I.** Graph depicting the fold increase change of each subset of Ht48 cells described in (H) after a 6-day culture in StemExpM. Each culture well is represented by a dot. Horizontal lines indicate the median fold increase change. **J.** Representative dot plots of a flow cytometric analysis to detect CD4, CD8, and c-kit expression in each subset of donor Ht48 cells described in (H) after a 6-day culture in StemExpM. **K.** A transplantation experiment with KDN cells was performed. The overall survival of recipient mice after receiving a transplantation of 5×10^3^ KDN cells or 1×10^6^ unfractionated Ht48 cells (Bulk) is shown. The overall difference in survival between these two groups is not statistically significant. **L.**-**M.** Graphs depicting the weight (L) and the cell number M of spleens after Ht48 cell or KDN transplantation. Each mouse is represented by a dot. Horizontal lines indicate the median. Ht48+, Ht48 cell transplantation; KDN, KDN transplantation; n.s., not significant. **N.** A representative dot plot from a flow cytometric analysis to detect CD4, CD8, and c-kit expression in donor Ht48 cells after transplantation of KDN.

To assess the role of SCF-c-kit signaling in ATLSC self-renewal, we first cultured 1×10^4^ Ht48 cells with or without stem cell expansion-inducing cytokines (StemExpM: TPO, IL-6, Flt3, IL-3, and SCF) or a T cell expansion-inducing cytokine (TcellExpM: IL-2) (Figure 5A) for 6 days. The total Ht48 cell number was not changed among these groups within the 6-day culture (Figure 5B), but only the StemExpM increased the percentage of c-kit^+^DN (KDN) cells (Figure 5C and 5D). These data suggest that only StemExpM preserves ATLSC renewal, *in vitro*.

We next cultured 1×10^4^ c-kit^+^ Ht48 cells with or without StemExpM for 6 days. As expected, the total cell number increased significantly more when the c-kit^+^ Ht48 cells were grown in StemExpM than in control medium (Figure 5E). Additionally, growth in the StemExpM induced more KDN cells than growth in control medium (Figure 5F). These data suggest that the c-kit^+^ ATLSCs were highly repopulated when the cells were grown in the StemExpM.

To understand which c-kit^+^ cell type contributes to Ht48 stem cell repopulation, we sorted Ht48 cells and cultured the KDN, c-kit^+^ DP (KDP), c-kit^+^4SP (K4SP), and c-kit^+^8SP (K8SP) cells in StemExpM (Figure 5H). The KDN population proliferated more than 50-fold in the StemExpM (Figure 5I and 5J). Together our findings suggest that the KDN population may contain ATLSCs, so we transplanted 5×10^3^ KDN Ht48 cells into Ly5.1 mice. As when 1×10^6^ Ht48 cells were transplanted, the mice that were transplanted with 5×10^3^ KDN Ht48 cells died within 40 days (Figure 5K) and the original ATL lymphoma was recapitulated in recipient mouse spleens (Figure 5L-5N).

### Molecular signature of KDN cells and SP cells

**Figure 6:**
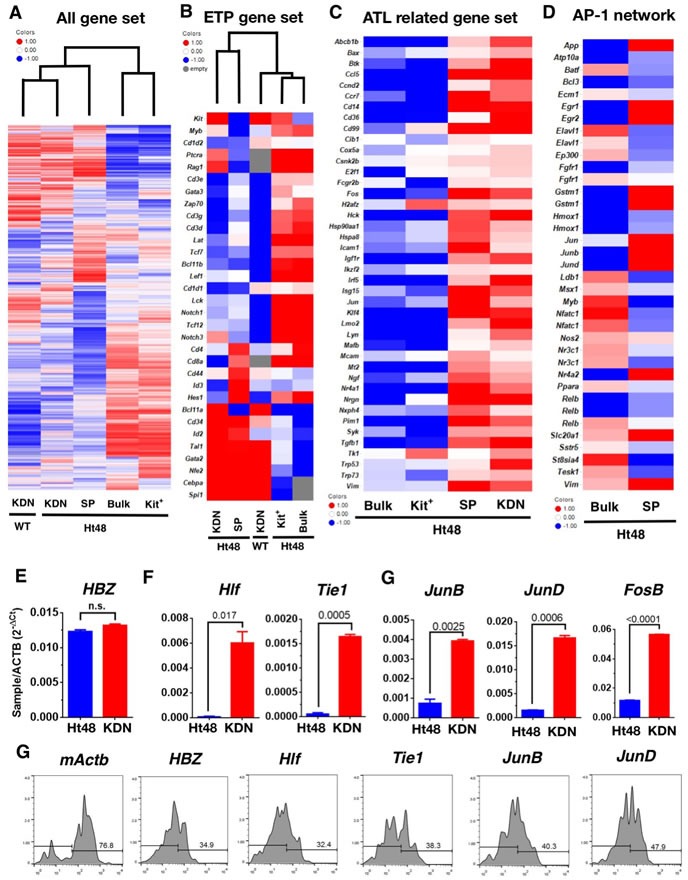
Global gene expression profile of Ht48 cells Ht48 cells were subdivided into c-kit^+^ cells, c-kit^+^CD4/8DN (KDN) cells, SP cells, and normal WT counterpart c-kit^+^CD4/8DN (WT-KDN) cells. **A.** Unsupervised hierarchal clustering of 15,882 gene expression profiles obtained from each fraction using an Agilent DNA microarray system. **B.** Hierarchal clustering of gene expression profiles obtained from each fraction using the ETP gene list. **C.** Gene expression profiles obtained from each fraction using the HTLV-1 infection and ATL-related gene list. **D.** Gene expression profiles obtained from each fraction using the AP-1 family gene network gene list. **E.**-**G.** A q-PCR analysis of differentially-expressed genes between unfractionated Ht48 cells (Bulk) and KDN cells. The expression levels of HTLV-1 HBZ **E.**, randomly selected genes from upregulated genes in SP cells, Hlf, Tie1, **F.**, and members of the AP-1 gene family including, Junb, Jund, Fosb in KDN **G.**. **H.** A flow cytometric analysis was performed using a Primeflow™ assay to detect q-PCR confirmed genes including, mActb, HBZ, Hlf, Tie1, Junb, and Jund in KDN cells. The Ht48 cells were separated by forward and side scatter (FSC and SSC, respectively) and by CD4^+^ and CD8^+^ for each gene transcript. Representative flow cytometry plots are shown, and the histograms depict the expression level of each gene in KDN cells.

Here, we phenotypically and functionally identified an ATLSC candidate, both *in vitro* and *in vivo*. To clarify the ATLSC candidate molecular signature, we performed a DNA microarray analysis of the SP, KDN, and c-kit^+^ Ht48 cell subsets as compared with normal KDN. Expression data for 28,305 genes were acquired, and the resulting heat-map shows partitioning of five samples and the 15,882 genes. A hierarchical clustering analysis clearly shows that there are two distinct Ht48 cell populations; the KDN and SP cells are different from the c-kit^+^ Ht48 cells and unfractionated Ht48 cells (Figure 6A). A similar clustering result was obtained in the immune regulatory genes (Supplemental Figure 1) and thymic early T-cell precursor (ETP) upregulated genes (Figure 6B). Among ETP gene set, we found HTLV-1 infection inducing gene, Nfe2 and Id2 upregulation both in the SP and KDN cells. Thus, we next analyzed the expression pattern of HTLV-1 and ATL related genes [29–34] in our mouse model. A similar clustering result was obtained in the ATL-related genes (Supplemental Figure 2). Among the 250 ATL-related genes, 43 genes, including, AP-1 family genes, Fos and Jun, were up regulated both in Ht48 SP cells and KDN cells (Figure 6C). We next analyzed the AP-1 gene family member expression patterns in our mouse model and found Jun, Junb, and Jund were upregulated in Ht48 SP cells (Figure 6D). We used q-PCR to confirm the gene expression levels of randomly selected (*Hlf*, *Tie1*) or AP-1 family genes that are differentially expressed in KDN cells. Whereas the HBZ expression level was not changed between the Ht48 cells and the ATLSC candidates (Figure 6D), the expression levels of *Hlf*, *Tie1*, *Junb*, *Jund*, and *Fosb* were upregulated in ATLSC candidates (Figure 6E-6G). Then, we used a Primeflow assay, which can detect mRNA expression, and found *mActb-*. *HBZ-*, *Hlf-*, *Tie1-*, *JunB-*, *JunD-*, expressing cells were detected among the KDN cells (Figure 6G). Finally, we listed the top 330 genes highly expressed in SP cells (Supplemental Table 3).

### c-kit-SCF signaling plays a key role in ATL progression *in vivo*

**Figure 7:**
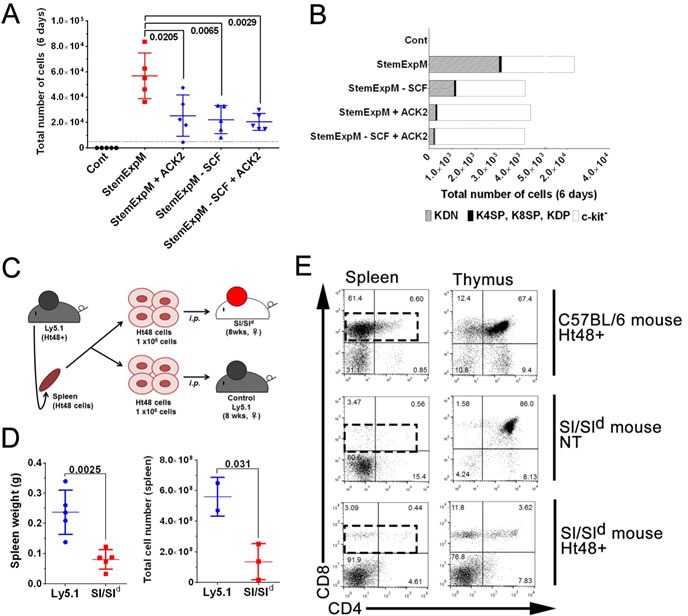
Effects of blocking SCF-c-kit signaling either *in vitro* or *in vivo* on ATLSC proliferation **A.** A total of 1×10^4^ Ht48 cells were cultured under a variety of culture conditions for 6 days. StemExpM: Stem cell expansion-inducing medium; StemExpM +ACK2: Stem cell expansion-inducing medium plus the c-kit-neutralizing antibody ACK-2; StemExpM -SCF: Stem cell expansion-inducing medium lacking SCF; StemExpM -SCF+ACK2: Stem cell expansion-inducing medium lacking SCF plus the c-kit-neutralizing antibody ACK-2. Graph depicting the number of MNCs in each culture condition. Each culture well is represented by a dot. Horizontal lines indicate the median number of MNCs. **B.** Percentage of KDN, c-kit^+^ cells excluding the CD4^−^CD8^−^ cells, and c-kit^−^ Ht48 cells after a 6-day culture under the conditions described in **A.**. **C.** Schematic representation of an *in vivo* transplantation experiment using Sl/Sld mice, which lack membrane-bound SCF. A total of 1×10^6^ Ht48 cells were transplanted intraperitoneally into control C57BL/6(Ly5.1) mice or Sl/Sld mice. **D.** Graph depicting the tissue weight (left panel) and the cell number (right panel) of each tissue after Ht48 cell transplantation. Each mouse is represented by a dot. Horizontal lines indicate the median value. **E.** Representative dot plots of a flow cytometric analysis to detect CD4 and CD8 expression in donor Ht48 cells in the spleen of recipient mice.

To confirm the role of c-kit-SCF signaling in ATLSC self-renewal, we blocked the c-kit signaling *in vitro* by neutralizing the c-kit receptor with the neutralizing antibody ACK2. Although StemExpM stimulates c-kit^+^ cell proliferation, ACK2 effectively blocked the c-kit^+^ cell proliferation as did StemExpM lacking SCF (Figure 7A). SCF neutralization *in vitro* also reduced ATLSC self-renewal (Figure 7B). The KDN Ht48 cell number dramatically reduced in StemExpM with ACK2 or lacking SCF.

To clarify the role of c-kit-SCF signaling *in vivo*, we transplanted 1×10^6^ Ht48 cells into Sl/Sld mice. These mice lack membrane-bound SCF, which is essential for hematopoietic stem cell proliferation and differentiation [35] (Figure 7C). Because most Sl/Sld mice die by 15 weeks old owing to severe anemia, we used 8 weeks old Sl/Sld mice and evaluated an engraftment of ATL cells 20-30 days after transplantation. No ATL development was detected in the recipient Sl/Sld mice. The spleen weight and MNC number in recipient Sl/Sld mice were lower than those in recipient Ly5.1 mice (Figure 7D). ATL cells, including 4SP, 8SP, and DP, were not observed in the Sl/Sld mouse spleens and thymuses (Figure 7E). These data suggest that c-kit-SCF signaling is essential for ATL development and progression *in vivo*.

## DISCUSSION

ATL is a malignant disease of mature T cells caused by HTLV-1 infection [1, 2] that has a poor prognosis owing to the resulting severe immune-suppression and its intrinsic resistance to chemotherapy [36]. Recently, the CSC hypothesis [37, 38] was proposed to explain the cause of ATL chemotherapy resistance [39]. Here, we found that ATLSC candidates display a c-kit^+^/CD4^−^/CD8^−^ phenotype in the HBZ-Tg ATL mouse model. ATLSCs have distinct phenotypic, genetic, and functional properties than the majority of ATL cells and can reconstitute the original ATL in recipient mice many times over and maintain a SP phenotype with a high drug efflux capacity [39]. These findings, together with our previous data, suggest that both Tax and HBZ might have the ability to induce leukemia stem cell properties in mouse T cells by inducing a high drug efflux ability.

The global gene expression study results indicate that many HBZ-interacting genes, such as the AP-1 family genes including *c-Jun*, *Junb* [40], and *Jund* [41], which are associated with leukemogenesis, are highly upregulated in ATLSCs. AP-1 family genes *c-fos*, *c-Jun*, *Atf,* and *Jdp* were also regulated by HTLV-1 Tax [42, 43], and they are involved in many cellular processes, such as proliferation, differentiation, and apoptosis. Interestingly, AP-1 and downstream microRNA-21 (miR-21) were upregulated in highly chemoresistant SP cells in several cell lines, and AP-1 inhibition reversed the chemoresistance and colony forming ability of SP cells [44]. Additionally, downregulation of junB/AP-1 transcription factor recapitulates the clinical aspects of CML [45] and AML [46] and JunB deficiency leads to a CSC-mediated myloproliferative disorder [47, 48]. Our microarray data show that *Junb* and *Jund* were also upregulated in the ATLSC fraction, and this finding was confirmed with the results from both q-PCR and FACS assays. Thus, both HBZ and Tax may induce or disrupt the AP-1 gene expression network and finally induce CSC properties, including the SP cell phenotype, *in vivo*. Further analyses are needed to confirm and reveal the molecular network underlining ATLSC development *via* the AP-1 gene family.

ATLSCs from both Tax- and HBZ-driven ATL commonly expressed c-kit, and the c-kit-SCF signaling pathway plays a major role in CSC-mediated ATL progression. Therefore, the SCF-c-kit signaling pathway is a possible therapeutic target in ATL. In mice, c-kit is a key regulator of hematopoietic stem and progenitor cells as well as testicular and intestinal cells [49]. Inhibition of c-kit kinase activity has been found several cancers. In AML, approximately 85% of AML cells express c-kit [50], and SCF combined with IL-3 stimulates massive AML cell proliferation [51]. Despite our previously published findings, it remained unknown if most ATL cells express c-kit. A distinct subset of self-renewing human memory CD8^+^ T cells with high dye efflux properties that expressed c-kit similarly to the Ht48 cells in our model has been reported [52]. Recent clinical studies also highlight the existence of ATLSCs in the ATL patient T memory stem cell fraction [53]. It is still unknown if T memory stem cells in ATL require SCF-c-kit signaling, and further functional studies are needed to determine if c-kit^+^ cells also possess CSC properties in human ATL cases.

When we compared ATLSCs with normal c-kit^+^CD4/8DN cell counterparts in a DNA microarray analysis, we found that each fraction showed a similar gene expression pattern suggesting that ATLSCs are of stem/progenitor cell origin or were reprogrammed into immature cell types by HTLV-1 infection.

In the thymus, immature T cell progenitors, DN1 cells [53], which express high levels of c-kit contain the most immature T lineage progenitors (ETPs) [54]. ETPs are derived from human stem cells but are non-renewing progenitors that have retained a limited multi-lineage differentiation potential. Oncogenically transformed ETPs might be one cause of recurrent leukemia, and they respond poorly to chemotherapy in T cell acute lymphoblastic leukemia [55]. In our analysis, the ETP gene set was also reclassified in HBZ-Tg ATL cells (Figure 6B) as well as in the global and immune gene sets. These data suggest that ATLSCs might acquire ETP properties or may maintain ETP properties during HTLV-1 infection. Because HTLV-1 infection occurs *via* breast feeding, and ETPs arise from the BM and are thought to migrate to the thymus during embryonic and postnatal development, ETPs may be a possible HTLV-1 infection target and long latency target for ATLSCs.

Recent studies suggest that human CD4/8DN cells may act as a reservoir of persistent of HIV-1 [56] and are associated with HIV-1 seroconversion [57]. Interestingly, CD4/8DN T cells still persist throughout highly active antiretroviral therapy suggesting that CD4/8DN T cells may affect drug resistance [58]. In addition, CD4/8DN T cells may play a variety of roles in the immune system, such as immune response, autoimmunity, immune tolerance in transplantation, and antitumor activity [59]. These data suggest that ATLSCs may play various roles in ATL development.

Although most of HBZ- expressing ATL cells (Ht48 cells) migrate into spleen like other T-ALL model [60], we found that HBZ-expressing ATL cells migrate and infiltrate into the ovaries (Figures 1 and 2). In non-Hodgkin lymphoma, although 7-25% of lymphoma cells infiltrate the ovaries [61], lymphomas arising primarily in extra-nodal sites (pENL) are extremely rare. Some pENL are associated with an underlying immunodeficiency syndrome, such as HIV/AIDS, or with an infection, such as Epstein-Barr virus, HTLV-1, human herpesvirus-8, or hepatitis C [62].

Most lymphoma cells are B cells, although some T cell and natural killer cell lymphomas were reported in the female genital tract [63]. In ATL, there are only a few reports that ATL cells infiltrate into the ovaries; many studies did not examine the ovarian involvement. Interestingly, an HTLV-1-inducible gene, TSCL1, promotes ovarian infiltration by an ATL cell line in the NOG mouse model [64]. Additionally, ATL-derived factor (ADF) has been detected in the human ovary throughout the menstrual cycle with high production levels during pregnancy [65]. ADF induces IL-2Ra in HTLV-1-infected cells [66] and promotes the proliferation of HTLV-1-infected cells. Because SCF is also produced in the ovaries [67] we speculate that both ADF and SCF might act as chemoattractive factors to entice HTLV-1-infected cells and ATLSCs. These data also indicate the ovaries, an immune-privileged organ [68], may be a possible new niche for HTLV-1-infected cells or ATLSC refuges during the long latency period.

## MATERIALS AND METHODS

### Mice

All mouse experiments were approved by the Animal Care and Use Committee of the National Institute of Infectious Disease, Tokyo, Japan. C57BL/6-Ly5.1 mice congenic for the CD45 locus were purchased from Sankyo-Labo. Sl/Sld mice (WBB6F1/kit-Kitsl /Kitsl-d/Slc) were purchased from SLC, Inc.

### Serial transplantation

We used Ht48 cells, which are splenic ATL cells established from HBZ-Tg mice [28], for the first transplantation. ATL-like lymphoma and leukemia was first established in a female, 6-8-week-old C57BL/6-Ly5.1 mouse by an intraperitoneal injection of 3.5×10^7^ frozen Ht48 cells. After 15 days, ATL-like lymphoma developed in the recipient mouse spleen, and the resulting Ht48 cells were capable of regenerating the original ATL-like lymphoma when further injected into new recipient mice. Using this transplantation system, Ht48 cells were serially passed as required.

### Cells preparation and flow cytometric analysis

The spleens, livers, bone marrow (BM), thymuses, ovaries, inguinal and axillary lymph nodes (LNs), and ovaries were removed from Ht48 cell-recipient mice. Monoculear cells (MNCs) were isolated from each tissue using Lymphoprep^TM^ (Axis-Shield), then stained with various antibodies (Supplemental Table 1) and with propidium iodide (50 ng; eBioscience) to distinguish live cells. For side population (SP) analysis, the MNCs were incubated (1 h; 37°C) with Hoechst33342 (5 μg/mL, Dojindo) with or without verapamil (25 μg/mL, Teva). Flowcytometric analyses and cell sorting with a J-SAN (Bay bioscience) were performed.

### Histological analysis

Tissues were fixed with Bouin's solution (Sigma-Aldrich) and then dehydrated using graded series of ethanol and cleared with xylen. Tissues were embedded in paraffin and sliced into approximately 4-μm-thick sections. We performed Periodic Acid-Schiff (PAS) hematoxylin staining and immunohistochemistry as previously described22. Primary antibodies (Supplemental Table 1) were detected with fluorescent-labeled secondary antibodies and nuclear DNA was detected with Hoechst33342. For single CD3 staining, a biotinylated secondary antibody was used with ABC solution (Vectastain) and colorized with diaminobenzidine (Merck Millipore). Sections were counterstained with hematoxylin. Microscopic analysis was performed with an Olympus BX53 and digital camera DP-73 with Cellsence capture and analyzing software.

### Cell culture

We cultured each Ht48 cell fraction with IL-3, SCF, IL-6 (20 ng/mL each), Flt3-L, and IL-3 (4 ng/mL each) in RPMI 1640 with 20% FBS for 6 days. All cytokines were obtained from Peprotech Inc (Supplemental Table 1).

### Microarray analysis

Total RNA was amplified and labeled with cyanine 3 (Cy3) using an Agilent Low Input Quick Amp Labeling Kit (Agilent). cRNA quantity and Cy3 incorporation were determined using a Nanodrop ND-1000 spectrophotometer and an Agilent 2100 Bioanalyzer. For each hybridization, 600 ng of Cy3-labeled cRNA were hybridized to an Agilent SurePrint G3 Mouse GE 8×60K Microarray (Design ID: 028005). Microarrays were scanned and the intensity values were quantified using Agilent feature extraction software, which performs background subtractions. Normalization was performed using Agilent GeneSpring (per chip: normalization to 75 percentile shift; per gene: normalization to the median of all samples). Data analysis and visualization including hierarchical clustering were carried out using TIBCO Spotfire (PerkinElmer).

### *in situ* hybridization

To detect the target gene transcripts in Ht48 cells, we performed flow-cytometric analyses combined with *in situ* hybridization analyses by Primeflow RNA assays (eBioscience) according to their protocols. For section *in situ* hybridization, we used viewRNA™ (eBioscience) according to their protocols. Target probes were designed with Affymetrix (Supplemental Table 1).

### q-PCR analysis

The primers for each target gene were designed with Primer 3 Plus (Supplemental Table 2). Total RNA (50 ng) was used for reverse transcription using SuperScriptIII First-Standard Synthesis System for RT-PCR (Thermo Fisher Scientific). Then, cDNA was amplified with SYBR-Premix-ExTaqII (Takara-Bio) and analyzed by Applied Biosystems 7500 Fast Real-Time PCR System. The resulting data were analyzed using the comparative CT value for relative gene expression quantification against the housekeeping gene, β-Actin (mouse Actb).

### Data analysis

All statistical analysis, including Student's *t*-test for gene expression analysis and Gehan-Breslow-Wilcoxon tests for survival curve analysis, were performed using GraphPad Prism (GraphPad Software) and Excel 2010 (Microsoft).

## SUPPLEMENTARY MATERIAL TABLES AND FIGURES




