# Supplementary material for: Impact of the SCF signaling pathway on leukemia stem cell-mediated ATL initiation and progression in an HBZ transgenic mouse model
Source: Oncotarget. 2016 Jun 21;7(32):51027–43. doi: 10.18632/oncotarget.10210 (PMC5239456; doi:10.18632/oncotarget.10210)
Supplement: Supplementary file 1 [file oncotarget-07-51027-s001.pdf]

Impact of the SCF signaling pathway on leukemia stem cell-mediated ATL initiation and progression in an HBZ transgenic mouse model

Supplementary Material

Supplemental Table 1: Antibodies, probes, and cytokines used in this study

Product name	Clone/CatNo	Application	manufcature
anti-CD4	RM4.5	FCM	eBioscience
anti-CD8a	53-6.7	FCM	eBioscience
anti-CD16/32	93	FCM	eBioscience
anti-CD38	90	FCM	eBioscience
anti-CD71	R17217	FCM	eBioscience
anti-CD117	2B8	FCM	BioLegend
anti-CD3	Ab5690	IHC	Abcam
anti-B220	RA3-6B2	IHC	eBioscience
anti-Ter119	TER-119	IHC	eBioscience
Donkey anti-Rat Alexa 488	17A2	IHC	Jackson Immuno Laboratories
Donkey anti-Rabbit - Cy3	RM4.5	IHC	Jackson Immuno Laboratories
HTLV-1 Tax	VF6-19283	ISH/FCM	Affymetrix
HTLV-1 Hbz	VF6-19284	ISH/FCM	Affymetrix
Hlf	VB1-17786	ISH/FCM	Affymetrix
Junb	VB1-17776	ISH/FCM	Affymetrix
Jund	VB1-17779	ISH/FCM	Affymetrix
Fosb	VB1-12485	ISH/FCM	Affymetrix
Tie1	VB1-17781	ISH/FCM	Affymetrix
mActb	VB1-10350	ISH/FCM	Affymetrix
c-kit	VB1-11509	ISH/FCM	Affymetrix
rMurine IL-2	212-12	CC	Peprotech
rMurine IL-3	212-13	CC	Peprotech
rMurine IL-6	216-16	CC	Peprotech
rMurine SCF	250-03	CC	Peprotech
rMurine Flt3-Ligand	250-31L	CC	Peprotech
rMurine TPO	315-14	CC	Peprotech

FCM: Flowcytometry, **IHC**: Immunohistochemistry, **ISH**: *in situ* Hybridization, **CC**: Cell Culture, **JIL**: Jackson Immuno Laboratories

Supplemental Table 2: Primers pairs used in this study.

Gene Symbol	forward	Reverse
HBZ	ctgtgcttgacggtttgcta	cctctttctcccgctctttt
Hlf	gctgggcaaatgcaagaaca	gacaggaaacaagctgtcca
Junb	gcctccgggacagtactttt	gggctttgacaaaaccgtcc
Jund	tcttgggctgctcaaactcg	tcgggtagaggaactgcgta
Tie1	agcagagtttgaagtgccca	tgacattgaccttgaagcgc
Fosb	tgcatcgaaacttgggcagt	atggccgagtggaatgagat
Klra4	acttacagcacacaggcaga	tggaattctggcagctctgt
Robo4	agaaaggtcgtggatgcagt	tagcgcattggttccctgtt
Klra7	attgccacgataactgcagc	agaagatcattgcctggcct
Cxcl16	aattggctggatgtcggcta	agcagcgccaacaagaaaag
Ndrg2	atagctacaacaaccgccga	acattccaccacggcatctt
mouseβ-actin	cagccttccttcttgggtatgg	ctgtgttggcatagaggtctttacg

Supplemental Table 3: List of up-regulated genes in Ht48 SP cells.

For Table S5, please see the attached Excel file

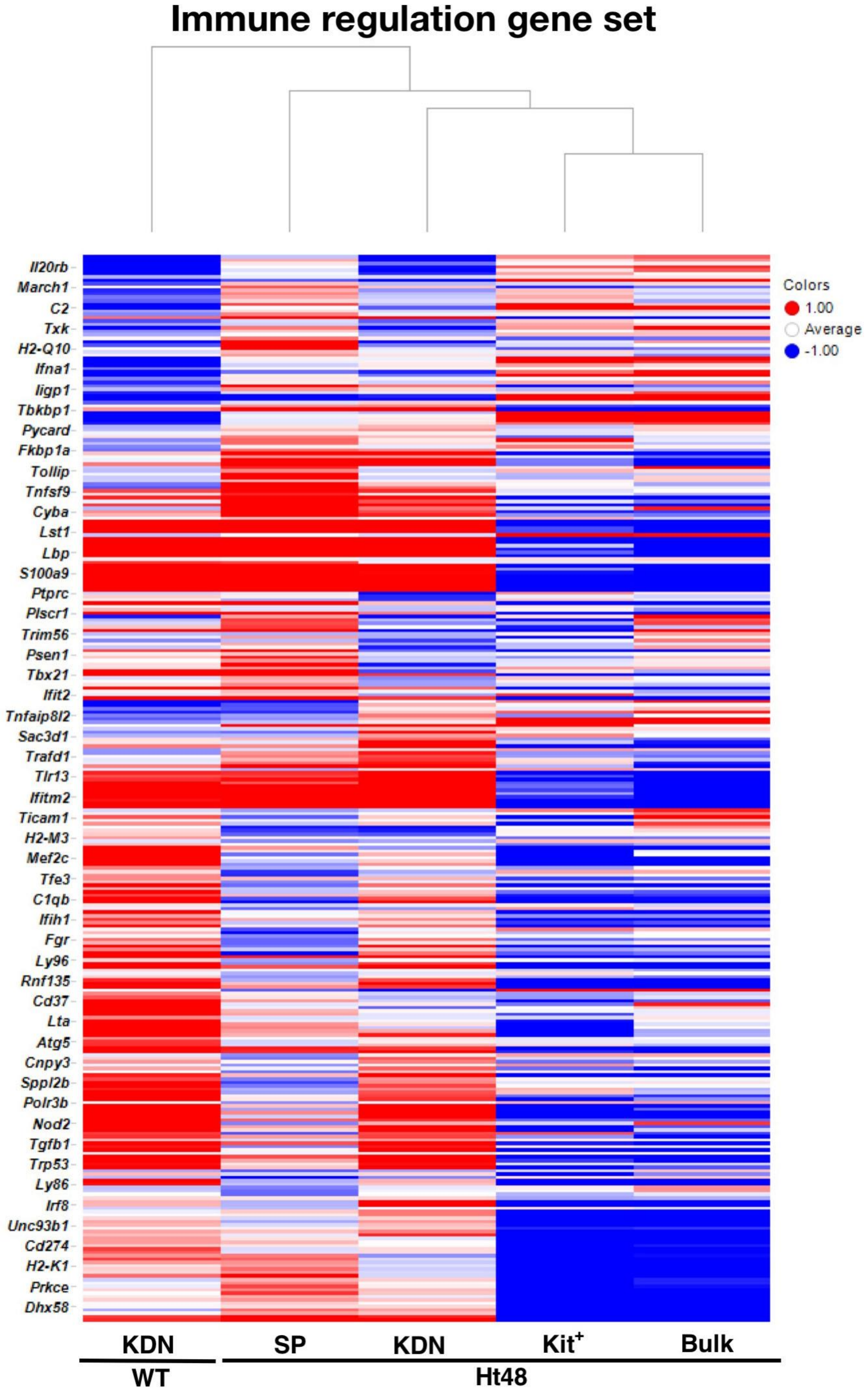
Immune regulation gene set
Il20rb
March1
C2
Txk
H2-Q10
Ifna1
Iigp1
Tbkbp1
Pycard
Fkbp1a
Tollip
Tnfsf9
Cyba
Lst1
Lbp
S100a9
Ptprc
Plscr1
Trim56
Psen1
Tbx21
Ifit2
Tnfaip8l2
Sac3d1
Trafd1
Tlr13
Ifitm2
Ticam1
H2-M3
Mef2c
Tfe3
C1qb
Ifih1
Fgr
Ly96
Rnf135
Cd37
Lta
Atg5
Cnpy3
Sppl2b
Polr3b
Nod2
Tgfb1
Trp53
Ly86
Irf8
Unc93b1
Cd274
H2-K1
Prkce
Dhx58
Colors
1.00
Average
-1.00
KDN
SP
KDN
Kit+
Bulk
WT
Ht48

Supplemental Figure 1. Global gene expression profile of Ht48 cells. Ht48 cells were subdivided into c-kit$^+$ cells, c-kit$^+$CD4/8DN (**KDN**) cells, SP cells, and normal WT counterpart c-kit$^+$CD4/8DN (**WT-KDN**) cells. Unsupervised hierarchal clustering of 632 immune regulatory gene expression profiles obtained from each fraction using an Agilent DNA microarray system. Immune regulatory genes were selected by the GO category.

ATL related gene set

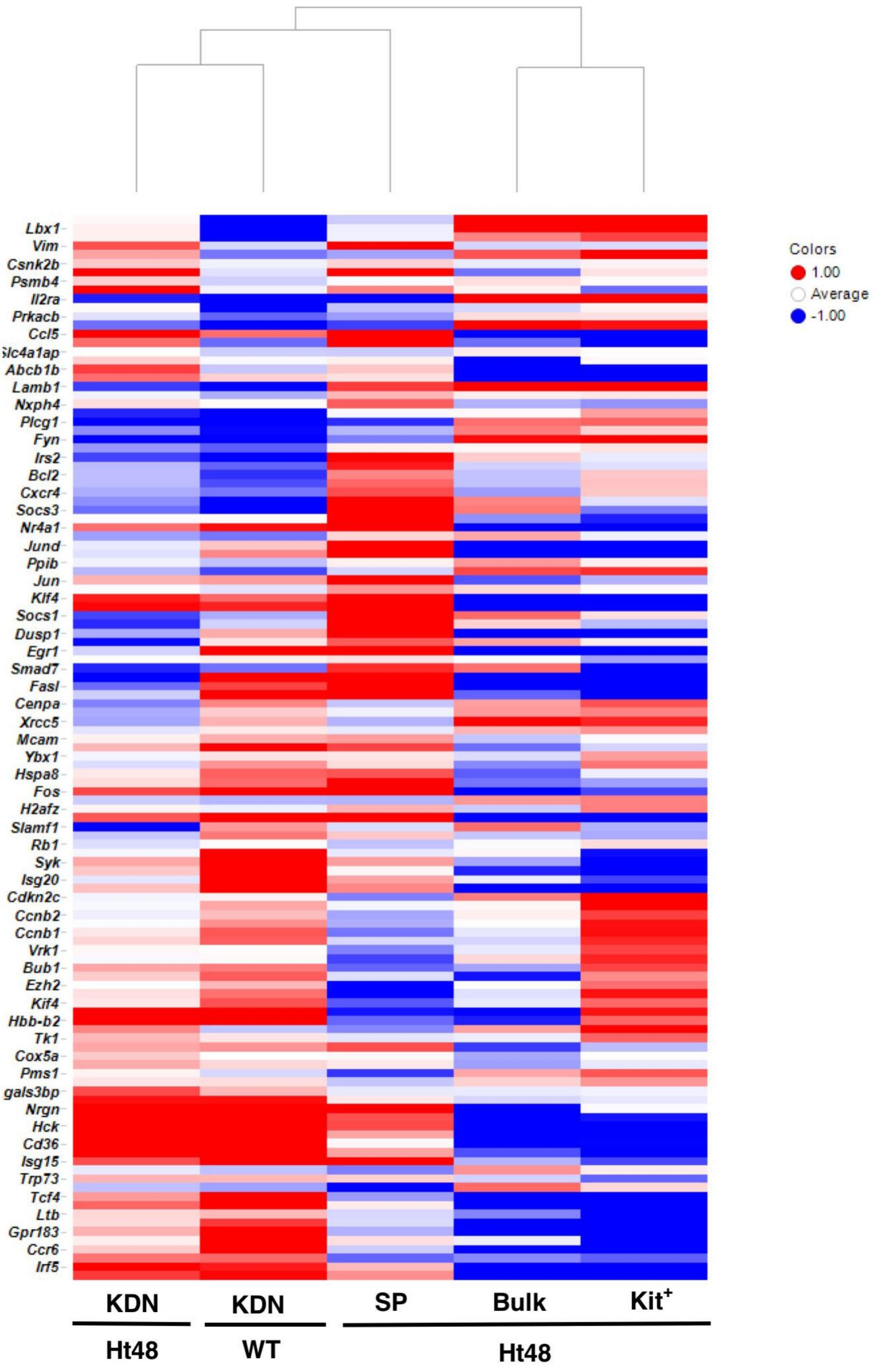

Supplemental Figure 2 Global gene expression profile of Ht48 cells. Ht48 cells were subdivided into c-kit$^+$ cells, c-kit$^+$CD4/8DN (**KDN**) cells, SP cells, and normal WT counterpart c-kit$^+$CD4/8DN (**WT-KDN**) cells. Unsupervised hierarchal clustering of 250 HTLV-1 infection- and ATL-associated gene expression profiles obtained from each fraction using an Agilent DNA microarray system.
